# Prevalence of lower second premolar agenesis and study of rhizolysis of primary second molars without permanent successor with 2 methods

**DOI:** 10.1007/s11282-025-00846-x

**Published:** 2025-08-13

**Authors:** María Lourdes García-Navas Fernández de la Puebla, Antonia María Caleya Zambrano, María Fe Riolobos González, Anabella Reyes Ortiz, Nuria Esther Gallardo López

**Affiliations:** 1 Faculty of Dentistry, Complutense University of Madrid, 28040 Madrid, Spain; 2 Dental Clinical Specialties Department. Faculty of Dentistry, Complutense University of Madrid, 28040 Madrid, Spain; 3 Master’s Degree in Pediatric Dentistry, Alfonso X El Sabio University, 28037 Madrid, Spain

**Keywords:** Root resorption, Infraocclusion, Primary second molar, Second premolar agenesis, Clinical pediatric dentistry

## Abstract

**Objectives:**

The aims of this study were to estimate the prevalence of lower second premolar (2Pr) agenesis and to quantify rhizolysis of the preceding primary molars.

**Materials and methods:**

A descriptive and cross-sectional study was carried out to quantify root resorption of lower primary second molar (p2m) with permanent successor agenesis in panoramic radiographs using 2 methods: Bjerklin–Bennett method (graphical method by fractions) and ratios’ method (proportions).

**Results:**

3206 panoramic radiographs were reviewed. The final sample consisted of 174 p2m. With the graphical method, it was found that half of the sample had both roots without resorption. With the numerical method, it was observed that root length decreased with age. Almost half of them have infraocclusion and greater infraocclusion led to greater resorption. Healthy p2m showed less resorption.

**Conclusions:**

The prevalence of 2Pr in the present study was 3.4%. The p2m with agenesis of the successor begin to resorb from the age of 9 years, remaining stable from this age. Those p2m without infraocclusion and healthy suffer less resorption. We consider both methods used to measure root resorption to be valid without the need for multiple sequential radiographs. We consider new studies necessary, expanding the sample and studying more variables that affect rhizolysis.

## Introduction

Under physiological conditions, tooth replacement involves the root resorption of primary teeth, leading to the loss of dental hard tissues, elimination of pulp, and periodontal ligament, along with alveolar bone remodeling, which may be influenced by general and local factors. This process is also called rhizolysis or rhizoclasia [1, 2].

Inheritance, endocrine disorders, nutritional deficits, and sex are some of the general factors that can affect dental resorption [1, 2]. Although the pressure from the permanent germ plays a crucial role in the root resorption, it also occurs in the presence of agenesis [2–5]. Additionally, factors, such as caries, fillings, infectious processes, pulp treatments, and infraocclusion of the primary tooth, have been associated with root resorption [2, 6, 7].

The lower primary second molar (p2m), like other molars, serves the function of chewing. Its distal aspect, along with its antagonist, establishes the terminal plane, that conditions the relationship of the permanent first molars. This role becomes more significant when there is agenesis of the successor, with a prevalence ranging from 2.4 to 4.3%, with 60% being bilateral presentation. A definitive diagnosis can be made at 9–10 years of age [8, 9].

As far as we have reviewed, methods used for studying root resorption of primary dentition have been graphical methods that analyze resorption through fractions [8–13]. Among them, the Bjerklin–Bennett method stands out, which was developed to study root resorption of p2m with agenesis of premolars’ successor. It consists of 6 stages, where the first stage represents absence of resorption, and the rest fractionate resorption by quarters [9]. Another notable method for studying rhizolysis is the one performed by Caleya et al. [14], which quantifies root resorption of lower primary molar through proportions (ratios), found by dividing both root lengths by the coronal height. The main advantage of this method is that it allows to quantify rhizolysis without the need for multiple sequential radiographs.

The objectives of this research were to estimate the prevalence of lower second premolar (2Pr) (absence of the p2m dental crypt of the permanent successor premolar) and quantify the degree of resorption of the preceding p2m depending on age, sex, infraocclusion, location, and dental health status (healthy/filled-carious) using a graphic method (Bjerklin–Bennett method) and a numerical method (ratios). Hence, the null hypothesis was that there is no association between root resorption of second lower primary molars with agenesis of the successor premolar and factors, such as time of remaining time in the mouth, infraocclusion, and health status (healthy deciduous molars or without treatments that do not interfere with the root resorption process), were included, sex, and location in the dental arch.

## Materials and methods

### Design and environment

A descriptive and cross-sectional study was conducted at the Integrated Children’s Clinic of Dentistry School at the Complutense University of Madrid (UCM). Also, STROBE guidelines (https://www.strobe-statement.org) were followed. Digital panoramic radiographs, performed from January 2007 to July 2021, were selected. The radiographic records had been made for diagnostic purposes unrelated to this study.

### Ethical aspects

This study was evaluated and accepted by the San Carlos Clinical Hospital’s Ethics and Clinical Research Committee, Madrid-Spain with reference: 22/692-E_Tesis. In addition, all patients (or guardians in the case of patients under 18 years of age) gave written consent to the use of their records and personal data by completing the informed consent form.

### Selection criteria

#### Inclusion criteria

Orthopantomographies belonged to patients aged between 6 and 21 years, with enough quality to correctly evaluate the molars under study, with the absence of the p2m dental crypt of the permanent successor premolar were selected.

#### Exclusion criteria

Panoramic digital radiographs belonging to patients with systemic diseases and/or syndromic conditions were excluded, as well as those with congenital orofacial malformations resulting in agenesis, alterations in eruption and rhizolysis, dental abnormalities affecting odontogenesis, local crown and/or root alterations, and those who had undergone or were undergoing orthodontic treatment. Additionally, p2m presenting extensive caries leading to significant coronal destruction, pathological dental wear, large reconstructions or full coverage crowns, pulp pathology, or any pulp treatment were also excluded.

### Sample size

The sample size was determined considering a 4.3% estimated prevalence and 3% accuracy by Epidat® version 4.2 software. The result was to consider at least 176 molars.

### Data collection procedures

The principal researcher was previously calibrated and trained, as well as being responsible for the selection and participant identification.

First, the patients were selected according to predefined inclusion criteria. In those cases, in which the patient had more than one radiograph with different dates, the data were taken into account as independent records. To carry out a more complete study and facilitate the study of the resorption, the sample was divided into different age groups: from 6 to 9 years (first phase mixed dentition); from 9 to 12 years (second phase mixed dentition); from 12 to 15 years and from 15 to 21 years, stages of puberty.

Second, the resorption pattern was identified by analyzing the radiographs.

For the assessment of root resorption using the graphical method, Bjerklin–Bennett method was employed, which establishes 6 stages and independently evaluates both roots (Stage 1: absence of root resorption; Stage 2: 1/4 root resorption; Stage 3: 1/2 root resorption; Stage 4: 3/4 root resorption; Stage 5: 4/4 root resorption; Stage 6: absence of the primary molar [9].

**Fig. 1:**
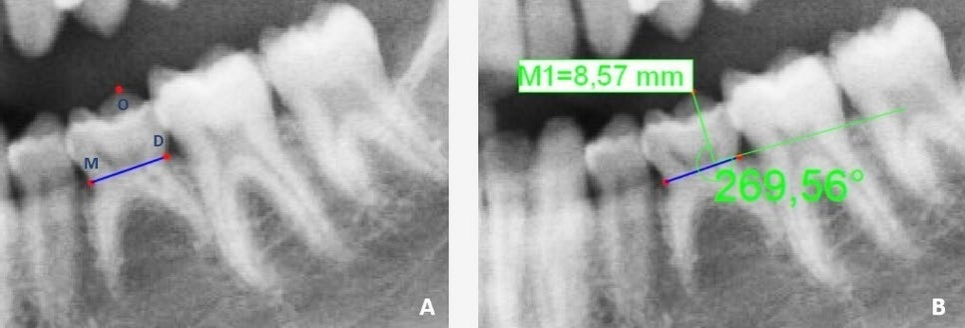
Coronal Height (M1). A: Anatomical points localization. B: Measurement from O to O-MD line. MD Line: Line connecting cementum- enamel junctions (mesial and distal) / O: Highest coronal point

**Fig. 2:**
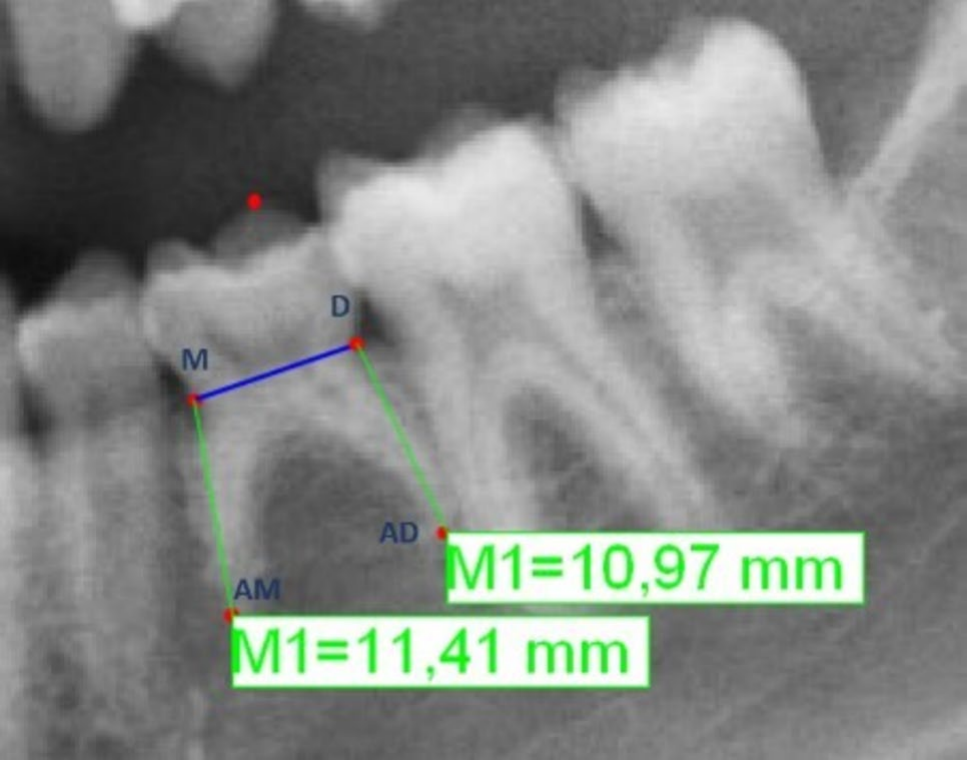
Measurement of Mesial and Distal Root Length (M1). M and D: Mesial and distal cementum-enamel junction, respectively. AM and AD: Most apical points of the mesial and distal roots, respectively

For evaluation root resorption using the numerical method, the method described by Caleya et al. [14] was utilized and a specific computer program (tps DIG 2®) was used. After locating both cementum–enamel junction (mesial and distal), a line connecting them (MD line) was drawn. The coronal height was determined by measuring the perpendicular line connecting the highest coronal point (O) with the MD line (Fig. 1). The mesial root length was measured from the mesial cementum–enamel junction (M) to the most apical point of the mesial root (AM). The distal root length: from the distal cementum–enamel (D) to the most apical point of the distal root (AD) (Fig. 2). Using linear measurements, the CCR were calculated for both mesial (CCR-m) and distal (CCR-d) roots, by dividing the length of each root by the coronal height.

**Fig. 3:**
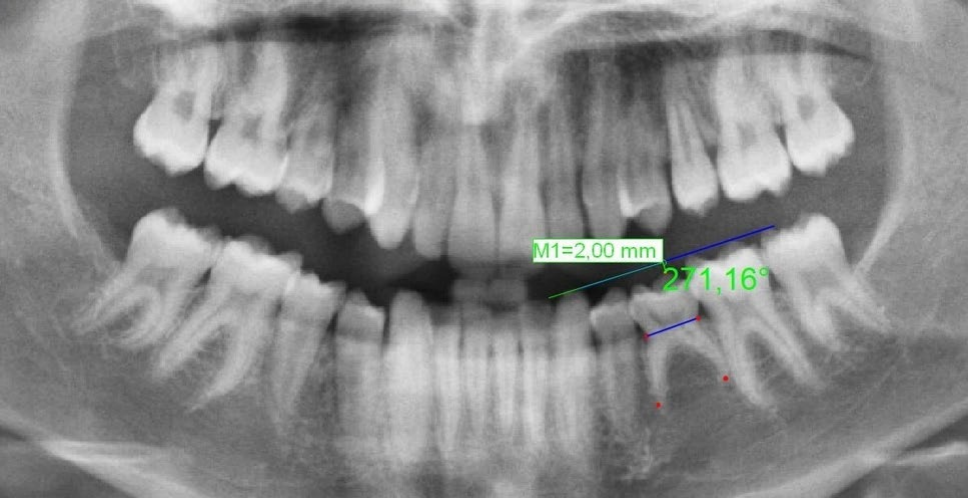
Infraocclusion Measurement

To assess infraocclusion, the method described by Cardoso et al. [8] (modified Bjerklin–Bennett method) was used. The occlusal plane established by the fully erupted permanent first molar occlusal surface was taken as reference (Fig. 3). Once the occlusal plane was marked, a perpendicular line was drawn from the highest coronal point, and then, the distance in millimeters from that point to the occlusal plane was calculated, thus determining the molar's infraocclusion. Infraocclusion values less than 1 mm were discarded, and three infraocclusion scores were established: mild, between 1.0 and 1.9 mm; moderate, between 2.0 and 2.9 mm; and severe, greater than 3 mm [8].

### Intra-examiner agreement

To determine intra-examiner agreement, a second measurement, 1 month after the first measurement, was performed on a total of 50 randomly selected samples, without knowledge of previously obtained data. It was found that, using the Chi-square test for categorical variables (p > 0.05) and the Student's t test for quantitative variables (*p* > 0.05), there were no significant differences for any of the variables.

### Statistical analysis

Descriptive statistics were conducted as percentages and confidence intervals.

For the comparison of categorical variables, the *Chi-square test* was applied. In cases where the criteria for conducting the *Chi-square test* were not met, such as an observed count less than 5 in 20% of the cells, *Fisher's exact test* was used.

For quantitative variables, asymmetry and kurtosis were studied, as well as the relevant normality test according to the recorded sample size (Kolmogorov–Smirnov or Shapiro–Wilk). For the comparison of quantitative variables, the independent samples *t test* was used within the framework of parametric tests. When comparing more than two groups, the *one-way ANOVA test* was employed followed by pairwise *post hoc tests*.

Statistical analysis was performed using the software application: IBM-SPSS Statistics version 26 (IBM Corp. Released 2019. IBM-SPSS Statistics for Windows, Version 26.0. Armonk, NY: IBM Corp).

## Results

### Prevalence

**Fig. 4:**
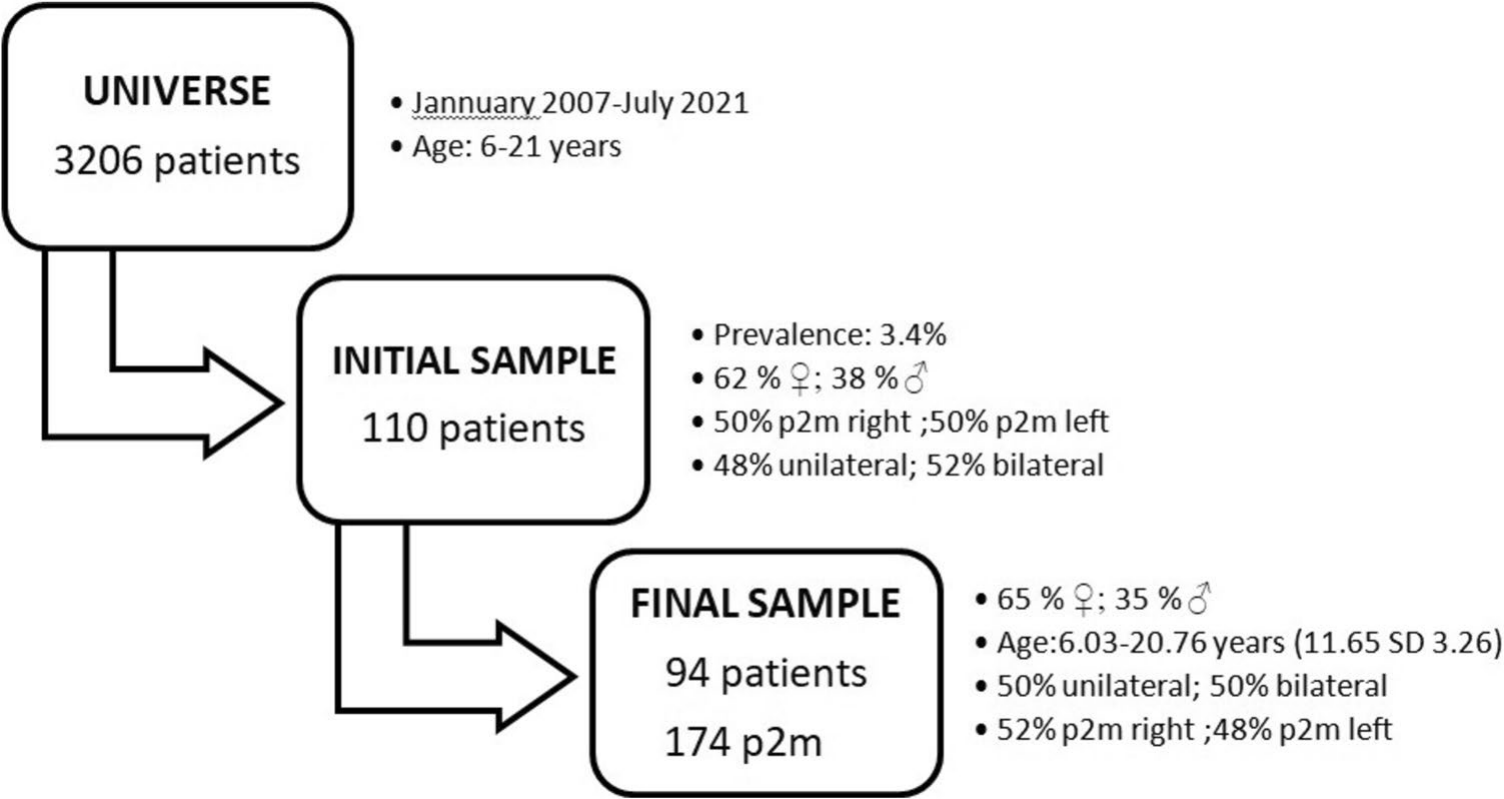
Flow diagram. Sample selection. p2m: Lower primary second molar

Digital panoramic radiographs belonging to 3206 patient records were reviewed. We found p2m with permanent successor agenesis in 110 patients, of whom 62% were female, 50% were left p2m, and 52% were bilateral agenesis. The prevalence of agenesis of the 2Pr in this study was 3.4% (Fig. 4).

To address our second objective and after applying the selection criteria, the final sample comprised 174 p2m from 94 patients (33 males and 61 females). The age ranged from 6.03 to 20.76 years (mean age: 11.65 years, SD 3.26). Fifty percent were bilateral agenesis, and 52.3% corresponded to the right p2m (Fig. 4).

### Assessment of resorption of p2m with successor agenesis vs age

When the association between the degree of root resorption and age was analyzed using the graphical method, without age segmentation, it was observed that 53.4% of mesial roots and 47.7% of distal roots were in stage 1 of Bjerklin–Bennett, that is, without resorption. Significant differences were found in both roots, with a small effect size (Table 1).

**Table 1:** Contingency table. Mesial and Distal Root Resorption (Bjerklin-Bennett graphical method) vs. age

Mesial root resorption	AGE(number of cases)				
	6–9 years	9–12 years	12–15 years	> 15 years	
Stage 1. No	71.7% (38)	50.8% (32)	56.7% (17)	21.4% (6)	53.4% (93)
Stage 2. 1/4	17.0% (9)	28.6% (18)	26.7% (8)	46.4% (13)	27.6% (48)
Stage 3. 2/4	5.7% (3)	17.5% (11)	16.7% (5)	21.4% (6)	14.4% (25)
Stage 4. 3/4	5.7% (3)	1.6% (1)	–	7.1% (2)	3.4% (6)
Stage 5. 4/4	–	1.6% (1)	–	3.6% (1)	1.1% (2)
	30.5% (53)	36.2% (63)	17.2% (30)	16.1% (28)	(174)

		*p* value	Statistical	Result	
	*χ*^2^	.015*	24.93	Significant	

		Value	Effect size		
	*V*	.219	Small		

Distal root resorption	AGE (number of cases)				
	6–9 years	9–12 years	12–15 years	> 15 years	
Stage 1. No	60.4% (32)	44.4% (28)	50.0% (15)	28.6% (8)	47.7% (83)
Stage 2. 1/4	24.5% (13)	33.3% (21)	13.3% (4)	32.1% (9)	27.0% (47)
Stage 3. 2/4	9.4% (5)	17.5% (11)	33.3% (10)	25.0% (7)	19.0% (33)
Stage 4. 3/4	5.7% (3)	4.8% (3)	3.3% (1)	14.3% (4)	6.3% (11)
Stage 5. 4/4	–	–	–	–	–
	30.5% (53)	36.2% (63)	17.2% (30)	16.1% (28)	(174)

		*p* value	Statistical	Result	
	*χ*^2^	.042*	17.44	Significant	

		Value	Effect size		
	*V*	.183	Small		

*NS* not significant, ^†^marginally significant (*p* < .10), *significant (*p* < .05), and **highly significant (*p* < .01) statistical test: *χ*^2^—Chi-square. Effect size: *V*—*V* Cramer's

When the association between the degree of root resorption and age was analyzed using the numerical method, for the mesial root, highly significant differences were found between the means of the group aged 6–9 years and the group over 15 years old, with a value of 0.218 (95% CI 0.061–0.374; *p* value = 0.002). For the distal root, highly significant differences were found between the means of the groups aged 6–9 years and 12–15 years, with a value of 0.227 (95% CI 0.069–0.384; *p* value = 0.001), and between the groups aged 6–9 and those over 15 years, with a value of 0.214 (95% CI 0.053–0.375; *p* value = 0.004).

Both CRR decrease very slightly as the age of the patients increases. From 9 years old, these remain unchanged significantly.

### Assessment of infraocclusion and its relationship with root resorption of p2ms with successor agenesis

It was found that 56.8% of the molars did not present infraocclusion, 25.8% were 1–2 mm, 12.3% were 2–3 mm, and 5.2% were greater than 3 mm. No statistically significant differences were found regarding sex. As infraocclusion increased, resorption also increased. When analyzed using the graphical method, significant differences were found between the groups (Table 2).

**Table 2:** Contingency table.Mesial and distal root resorption (Bjerklin-Bennett graphical method) vs. infraocclusion measured in mm

Mesial root resorption	Infraocclusion (number of cases)				
	> − .99	− 1.00 a − 1.99	− 2.00 a − 2.99	< − 3.00	
Stage 1. No	64.8% (57)	45.0% (18)	26.3% (5)	–	51.6% (80)
Stage 2. 1/4	23.9% (21)	42.5% (17)	36.8% (7)	12.5% (1)	29.7% (46)
Stage 3. 2/4	8.0% (7)	10.0% (4)	26.3% (5)	87.5% (7)	14.8% (23)
Stage 4. 3/4	1.1% (1)	2.5% (1)	10.5% (2)	–	2.6% (4)
Stage 5. 4/4	2.3% (2)	–	–	–	1.3% (2)
	56.8% (88)	25.8% (40)	12.3% (19)	5.2% (8)	(155)

		*p* value	Statistical	Result	
	*χ*^2^	.000**	54.80	Highly significant	

		Value	Effect size		
	*V*	.343	Medium		

Distal root resorption	Infraocclusion (number of cases)				
	> − .99	− 1.00 a − 1.99	− 2.00 a − 2.99	< − 3.00	
Stage 1. No	50.0% (44)	42.5% (17)	42.1% (8)	–	44.5% (69)
Stage 2. 1/4	28.4% (25)	35.0% (14)	26.3% (5)	–	28.4% (44)
Stage 3. 2/4	18.2% (16)	17.5% (7)	15.8% (3)	87.5% (7)	21.3% (33)
Stage 4. 3/4	3.4% (3)	5.0% (2)	15.8% (3)	12.5% (1)	5.8% (9)
Stage 5. 4/4	–	–	–	–	–
	56.8% (88)	25.8% (40)	12.3% (19)	5.2% (8)	(155)

		*p* value	Statistical	Result	
	*χ*^2^	.001**	29.34	Highly significant	

		Value	Effect size		
	*V*	.251	Small		

*NS* not significant, ^†^marginally significant (*p* < .10), *significant (*p* < .05), and **highly significant (*p* < .01) statistical test: *χ*^2^—Chi-square. Effect size: *V*—Cramer's *V*

Regarding the numerical method, significant differences were found between the means of CRR-m between the > -0.99 mm infraocclusion group and the < -3.00 mm infraocclusion group, with a mean difference of 0.257 (95% CI 0.17–0.497; *p* = 0.030).

Similarly, these results were reproduced in the CRR-d, although in this case, highly significant differences were found between the > 0.99 mm infraocclusion group and the < -3.00 mm infraocclusion group with a mean difference of 0.352 (95% CI 0.104–0.600; *p* = 0.002), and significant differences between the -1.00 mm to -1.99 mm and the < -3.00 mm infraocclusion groups with a mean difference of 0.307 (95% CI 0.047–0.567; *p* = 0.013).

### Assessment of resorption of p2ms with successor agenesis vs health status, sex, and location in the dental arch

Molars with healthy status presented less resorption, finding significant differences for both roots with the graphical method (Table 3).

**Table 3:** Contingency table. Variables:mesial and distal root resorptin (Bjerklin-Bennet graphical method) versus dental status

Mesial root resorption	2 pm				*p* value	Statistical	Result
	Healthy	Caries					
Stage 1. No	58.6% (82)	32.4% (11)	53.4% (93)	*χ*^2^	.000**	22.20	Highly significant
Stage 2. 1/4	28.6% (40)	23.5% (8)	27.6% (48)		Value	Effect size	
Stage 3. 2/4	10.7% (15)	29.4% (10)	14.4% (25)	*V*	.357	Medium	
Stage 4. 3/4	2.1% (3)	8.8% (3)	3.4% (6)				
Stage 5. 4/4	–	5.9% (2)	1.1% (2)				
	80.5% (140)	19.5% (34)	(174)				

Distal root resorption	2 pm				*p* value	Statistical	Result
	Healthy	Caries					
Stage 1. No	52.1% (73)	29.4% (10)	47.7% (83)	*χ*^2^	.003**	14.25	Highly significant
Stage 2. 1/4	28.6% (40)	20.6% (7)	27% (47)	V	Value	Effect size	
Stage 3. 2/4	15% (21)	35.3% (12)	19% (33)		.286	Small	
Stage 4. 3/4	4.3% (6)	14.7% (5)	6.3% (11)				
Stage 5. 4/4	–	–	–				
	80.5% (140)	19.5% (34)	(174)				

*NS* not significant, ^†^marginally significant (*p* < .10), *significant (*p* < .05), and **highly significant (*p* < .01) statistical test: *χ*^2^—Chi-square. Effect size: *V*—Cramer's *V*

Lower root resorption was observed in male individuals, with significant differences found in the distal root (Table 4).

**Table 4:** Contingency table. Variables: mesial and distal root resorption (Bjerklin-Bennet graphical method) versus sex

Mesial root resorption	Sex				p value	Statistical	Result
	Male	Female					
Stage 1. No	63.9% (39)	47.8% (54)	53.4% (93)	*χ*^2^	.135^NS^	7.02	Not significant
Stage 2. 1/4	24.6% (15)	29.2% (33)	27.6% (48)		Value	Effect size	
Stage 3. 2/4	9.8% (6)	16.8% (19)	14.4% (25)				
Stage 4. 3/4	–	5.3% (6)	3.4% (6)				
Stage 5. 4/4	1.6% (1)	.9% (1)	1.1% (2)				
	35.1% (61)	64.9% (113)	(174)				

Distal root resorption	Sex				p value	Statistical	Result
	Male	Female					
Stage 1. No	60.7% (37)	40.7% (46)	47.7% (83)	*χ*^2^	.001**	.17.57	Highly significant
Stage 2. 1/4	11.5% (7)	35.4% (40)	27.0% (47)		Value	Effect size	
Stage 3. 2/4	26.2% (16)	15.0% (17)	19.0% (33)	*V*	.318	Medium	
Stage 4. 3/4	1.6% (1)	8.8% (10)	6.3% (11)				
Stage 5. 4/4	–	–	–				
	35.1% (61)	64.9% (113)	(174)				

*NS* not significant, ^†^marginally significant (*p* < .10), *significant (*p* < .05), and **highly significant (*p* < .01) statistical test: *χ*^2^—Chi-square. Effect size: *V*—Cramer's *V*

With the numerical method, significant differences were found for the CRR-m according to sex, with higher ratios observed in males. Healthier molars exhibit higher CRR in both roots (Table 5).

**Table 5:** Contrast t-student/post hoc (Tukey). Means differences. Variables: CRR-m y CRR-d versus sex and health status

Variable	Group 1	Group 2	Means dif.	IC 95%		Sig.
				Up lim.	Low lim.	
CRR-m	Male	Female	.095	.012	.178	.025*
	Healthy	Caries	.171	− .073	.269	.001**
CRR-d	Male	Female	.054	− .032	.142	.215^**NS**^
	Healthy	Caries	.158	.056	.261	.003**

*NS* not significant, ^†^marginally significant (*p* < .10), *significant (*p* < .05), and **highly significant (*p* < .01)

Regarding the location in the arch, no statistically significant differences were observed with any method used.

When comparing root resorption of both p2m in cases of unilateral 2Pr agenesis with the contralateral, it was found that, in the mesial root, in the age groups of 6–9 years, and in the total sample, there is a higher frequency of coincidence in stages 1 (no resorption) and 3 (2/4 resorption) than in other stages and age groups. In the distal root, in children aged 6–9 years and in the total sample, the percentage of coincidence of stages of resorption 1, 2, and 3 is significantly higher. Both CRR have a moderate correlation.

## Discussion

There are different types of studies to analyze the resorption of primary molars, although not many are performed when there is agenesis of the successor premolar. In the case of dental agenesis, notable studies include those of Bjerklin and Bennet [9], Rune and Särnas [15], Kurol and Thilander [16], Ith-Hansen and Kjaer [17], Nordquist et al.[18] and Hvaring and Birkeland [19].  Ideally, a longitudinal prospective study should be performed [10, 15–17, 19], but this requires periodic radiographs for research purposes, which is not accepted by current radiation protection protocols [20, 21]. Therefore, the present study is cross-sectional and retrospective, a methodology validated by multiple previous studies [18, 22–24].

For sample selection, the criteria applied in this study are similar to those of other authors, such as Haselden et al. [23] or Calheiros-Lobo et al. [25]. The minimum age chosen was 6 years, since, according to previous studies, resorption of the 2Pr begins around that age [11, 13, 26, 27]. However, other authors point out that a precise diagnosis of 2Pr agenesis cannot be made with certainty until the age of 9–10 years, as these teeth may calcify late [28].

We aim to provide qualitative and quantitative values for root resorption. Similar to our study, orthopantomography has been the complementary test used by other authors in their studies for making measurements, especially when studying the molar region, where the distortion that can be presented by radiography is minimal [16, 17, 19, 22–25, 29]. Also, we have used panoramic radiographs, as they are used in pediatric dentistry as diagnostic records.

Regarding premolar agenesis, in the Spanish population, Vaquero-Niño et al. [30] estimated a prevalence of 4.9%, while Hernández Guevara [31] reported 4.1%. More specifically, when we talk about 2Pr, in our study, the prevalence of agenesis drops to 3.4%, with 50% of cases being bilateral. These results coincide with those offered by other authors, who report a prevalence ranging from 2.4 to 4.3%, with 60% of cases being bilateral [9, 10, 32]. However, other studies indicate that unilateral 2Pr agenesis is more common than bilateral [33] Regarding sex, in our study, the number of women with agenesis was almost double that of men (33 men and 61 women), consistent with eight of the studies reviewed [9, 10, 15, 22–25, 29].

Finally, the present study comprised 174 p2m from 94 patients. Reviewing the literature, we observed great variability in sample sizes. The study presented by Hvaring et al. [24], where they studied 188 p2m from 111 patients, resembles the sample size of this study.

To measure rhizolysis, the graphical method described by Bjerklin–Bennett in 2000 was used [9]. This method has been employed in other studies for analyzing root resorption in cases of agenesis [10, 18, 19, 24, 25]. The results differ from those; of Bjerklin and Bennett [9] and Bjerklin et al*.* [10]. However, they resemble those of Hvaring et al*.* [24], because both studies used orthopantomograms and had very similar data for healthy and filled molars.

When analyzing root resorption using the graphical method and its relationship with sex, no significant differences were observed for the mesial root. However, highly significant differences were found for the distal root, with a higher number of unresorbed roots in men. There was a greater difference in women when root resorption was 1/4. Other authors who used graphical methods to analyze resorption found no relationship between sex and resorption of both mesial and distal roots [15, 18, 19, 23]. We also found no differences when analyzing mesial root resorption using the numerical method, but we did find differences for the distal root.

Along with the graphical method, which can sometimes be subjective, we have used a numerical method that allows comparison of linear measurements with non-variable structures (coronal height) and minimizes phenomena, such as magnification, head position, differences between sexes, or racial differences. We use a numerical method based on ratios or proportions which was used to measure root resorption of p2m, but without agenesis [14].

Comparing the results with those of Caleya et al. [14], the mean ratio for the age range of 6–9 years in this study resembles theirs for both roots. This could be interpreted as similar resorption in this age group regardless of the presence or absence of dental germ. For the age group of 9–12 years, the ratio in this study is higher, indicating a delay in root resorption in cases of agenesis compared to resorption in the presence of a germ. The mesial and distal ratios for men are higher than those for women, very similar to the results of Caleya et al. In their study, they also indicated that there were differences in the ratio by sex. However, when we analyzed the distal root by ratios, we did not find statistically significant differences in terms of sex. The fact that the ratio for males is higher than that for females could indicate that the roots of males undergo slower resorption, which would coincide with the results of the graphical method for the distal root.

Since we do not coincide either in the method used or in the classification of infraocclusion, we cannot make direct comparisons with other authors.

Studies that analyze root resorption in cases of agenesis of the permanent successor, with the exception of Haselden et al. [23] examined how infraocclusion influences them. However, not all of them used the same method to assess infraocclusion, nor the same classification as we did in this study, making it difficult to make direct comparisons with our results. Rune and Särnas [15] found no relationship between infraocclusion, sex, caries and resorption, as did Nordquist et al*.*[18]. Kurol and Thilander stated that the progression of infraocclusion of those molars with agenesis is greater than when the germ is present [16]. Hvaring et al*.* [24] like Garib et al*.*[22] found a relationship between infraocclusion and root resorption, with infraocclusion and resorption increasing with age.

Another variable analyzed was whether the resorption of the p2m with agenesis of the successor could be altered when it suffered from any pathology. It has been observed that, with both methods, healthy p2m undergo less resorption than those with fillings or decay. In this aspect, we differ from Rune and Särnas [15] and Nordquist et al*.*[18] as they stated that there was no relationship between resorption and the presence of cavities or restorations.

Regarding comparing the resorption of p2m with agenesis of the successor with the contralateral without agenesis, as far as we have reviewed, we have not found any publication that analyzes it. There are studies that claim that when there is agenesis of the successor, resorption occurs more slowly or starts later [3, 4, 11, 12, 34, 35]. However, in our study, due to the sample size available to us, although we have compared all cases of unilateral agenesis, we cannot affirm that there are differences in the resorption pattern.

We are aware that our study has a significant limitation in that it is a cross-sectional study. A longitudinal study would be the ideal design to study a process that occurs over time, but multiple sequential radiographs of participants would be required, which is ethically controversial.

One of the greatest strengths of our study is the large sample size achieved to calculate the prevalence of second premolar agenesis. Another strength to highlight is that the previous studies only used graphical methods for analyzing resorption, whereas we used and compared two methods. Another aspect to emphasize is the large number of radiographs analyzed, all performed with the same X-ray machine. With this research, we aim to provide data to predict the survival of a molar with agenesis of permanent successor without the need for multiple sequential radiographs.

## Conclusion

The prevalence of 2Pr in the present study was 3.4%. The p2m with agenesis of the successor begin to resorb from the age of 9 years, remaining stable from this age. Those p2m without infraocclusion and healthy suffer less resorption. We consider both methods used to measure root resorption to be valid without the need for multiple sequential radiographs. We consider new studies necessary, expanding the sample and studying more variables that affect rhizolysis.

## Data Availability

Data will be made available on request.

## Abbreviations

p2m: Lower primary second molar
2Pr: Lower second premolar
MD Line: Line connecting the mesial and distal cementum–enamel junction, respectively
O: Highest coronal point
M: Mesial cementum-enamel junction
D: Distal cementum-enamel junction
AM: Most apical point of the mesial roots
AD: Most apical points of the distal roots
CRR: Crown-to-root ratio
CCR-m: Mesial crown-to-root ratio
CCR-d: Distal crown-to-root ratio
OPG: Orthopantomography
